# The risk of reverse zoonotic transmission to pet animals during the current global monkeypox outbreak, United Kingdom, June to mid-September 2022

**DOI:** 10.2807/1560-7917.ES.2022.27.39.2200758

**Published:** 2022-09-29

**Authors:** Wendi Shepherd, Philippa M Beard, Sharon M Brookes, Andrew Frost, Helen Roberts, Katherine Russell, Steve Wyllie

**Affiliations:** 1 Emerging Infections and Zoonoses Team, UK Health Security Agency, London, United Kingdom; 2 The Pirbright Institute, Pirbright, Surrey, United Kingdom; 3 One Health/Animal and Zoonotic Viral Diseases Portfolio, Animal and Plant Health Authority, Addlestone, Surrey, United Kingdom; 4 Department for Environment, Food, and Rural Affairs, London, United Kingdom

**Keywords:** Monkeypox, pets, reverse transmission, zoonosis, poxvirus, one health

## Abstract

We report results of surveillance between June and mid-September 2022 of pet animals living in households of confirmed human monkeypox (MPX) cases. Since surveillance commenced, 154 animals from 40 households with a confirmed human MPX case were reported to the United Kingdom Animal and Plant Health Agency. No animals with clinical signs of MPX were identified. While a risk of transmission exists to pets from owners with a confirmed MPX virus infection, we assess this risk to be low.

From May 2022 up to 16 September 2022, there have been 3,439 confirmed cases of monkeypox (MPX) in the United Kingdom (UK) linked to the current global MPX (Clade IIb, formerly West African clade) outbreak [1]. The recent rapid increase in the number of human MPX cases has led to questions about the risk of reverse zoonotic transmission from human cases to animals and the potential for establishing monkeypox virus (MPXV) in an endemic reservoir animal species [2], but the current risk of reverse zoonotic transmission is unclear. 

We performed 3.5 months of surveillance (1 June–16 September 2022) of pet animals cohabitating with a confirmed human MPX case. Here, we outline the current UK risk assessment and the surveillance system, and discuss the implications of surveillance findings on the future MPX outbreak response.

## Current surveillance system

In May 2022, as part of the initial UK response to the current global MPX outbreak, a standing multi-agency group of animal and public health experts (the Human Animal Infections and Risk Surveillance Group (HAIRS)) reviewed the current evidence of the risk ‘posed by mammalian pets exposed to monkeypox virus to people with whom they may come into contact’ [3]. The HAIRS group conducted a risk assessment and determined there was a limited evidence base in relation to the susceptibility of non-rodent pets to MPXV and advised a ‘precautionary risk management approach’. For example, for rodents, a known MPXV reservoir for whom the transmission risk has been established [4], this approach could include temporarily moving the rodent pet from the household during the recommended 21-day quarantine (based on incubation period of MPXV in humans) [5].

Based on the HAIRS assessment, on 1 June 2022 the UK Health Security Agency (UKHSA) and Animal and Plant Health Agency (APHA) implemented a surveillance system to assess for MPX in all animals residing within the households of confirmed human MPX cases. The purpose of this surveillance was to provide advice on how to care for the animal during the infectious period of the human case, which included the option for quarantine of rodent pets, and to ascertain if the animal was displaying any potential signs of MPX so that appropriate diagnostics and supportive treatment could be offered to the pet. Surveillance by APHA will continue while there is evidence of human-to-human transmission of MPX in the UK. 

## Individual risk assessment and guidance

In the initial stages of the MPX outbreak (1 June–7 July 2022), and as part of routine UKHSA investigations, confirmed human cases were explicitly asked about the number and type of any animals in the household. This information was passed directly to APHA so they could contact the case for a risk assessment, provision of appropriate advice and arrange quarantine of the pet, if indicated. On account of increasing human case numbers, from 7 July 2022, all MPX cases were asked to self-report information about household pets via an online UKHSA questionnaire and consequently received appropriate APHA guidance electronically, including information on how to access veterinary support if they were concerned about illness in their pet within 21 days from date of last exposure of the animal to the case, while the case was infectious [6]. All pet animals resident at the home of a confirmed MPX case, reported to APHA by either UKHSA, private veterinarian or owner from 1 June 2022 onwards, are included in our surveillance data. 

Since late June, all households with a confirmed human MPX case were provided with information from APHA at the time of initial diagnosis about how to care for pets and when to seek veterinary input online [7]. UKHSA health protection teams (HPTs), who provided the case and their contacts with infection prevention advice, and veterinarians could also contact APHA at any point after diagnosis of the human MPX case for advice about any animal if the case raised concerns about their pet. APHA was available to provide bespoke advice, liaise with the owner’s private veterinarian to undertake appropriate assessment of an animal with symptoms of MPXV infection, and, if unable to rule out infection, potentially arrange for MPXV testing. APHA is the sole competent authority-approved testing facility for MPX in animals in the UK. If infection were to be confirmed in a mammalian pet, the UK Department for Environment, Food and Rural Affairs (Defra) would inform the World Organisation for Animal Health as required, under our international obligations [8].

## Surveillance outcomes

Our 3.5-month surveillance, combined with observations from May 2022, showed that there have been no cases of animals with clinical signs suggestive of MPXV infection associated with confirmed human cases reported to APHA since the start of this MPX outbreak within the UK up to 16 September 2022.

Since the implementation of specific questioning of human cases regarding pet ownership on 1 June 2022, there have been 154 pets owned by confirmed MPX cases (n = 40) directly reported to APHA by HPTs following initial case investigations. Of these animals, 42 were dogs and 26 were cats (see Supplementary File S1 for complete list of reported animals by household). Advice and guidance were provided to these pet owners. To our knowledge, no animals have been presented to a veterinary practice, placed into quarantine, or tested for MPXV in the UK at the time of publication.

## Discussion

Monkeypox is a zoonotic disease caused by the orthopoxvirus MPXV. The MPXV is believed to be endemic in wild rodent populations in forested areas of central and western Africa [9]. This is of significance because mammals – particularly rodents – have previously been documented as reservoirs of poxviruses from animals to humans [10-16]. A recent report from France suggested a potential case of reverse zoonotic transmission of MPXV to a dog during the current MPX outbreak [17]; the animal tested positive for MPXV by cutaneous swab but negative by serology [18]. A single case of reverse transmission to a dog has also been reported in Brazil [19]. The reported cases of MPX in dogs suggest that there is the potential for reverse zoonosis transmission of MPXV (Clade IIb). Other domestic animals may also be at risk of MPX. While these reports did not change the HAIRS risk assessment, such findings reduce the uncertainty around the susceptibility of non-rodent mammalian pets.

In the UK, pet ownership is considered high with around 10 million each of cats and dogs owned by a population of 28 million households [20]. Based on the number of confirmed cases of MPX in the UK by 16 September 2022 (n = 3,439), we would crudely estimate 1,200 of these cases to live with pets. This is a straight-line calculation that does not adjust for demographics of MPX cases against demographics of pet owners in the UK, or any other variable. However, this estimate suggests that there is substantial under-reporting of pet ownership associated with MPX cases. 

We believe that there are several factors responsible for the likely under-reporting of pets associated with confirmed cases, which may introduce bias. Firstly, limited disclosure by pet owners could have impacted the surveillance outcome. At the start of the outbreak, the initial HAIRS risk assessment was widely reported by the media with a focus on the recommendation of quarantine for pet animals of MPX cases, although the HAIRS assessment only recommended quarantine for rodent pets. This may have caused to pet owners not to disclose pet ownership, or not to report animals with clinical signs, for fear of their pet being removed from the home. Secondly, there may have been incomplete transfer of information about pet ownership from HPTs to APHA. This was a manual process reliant on HPTs identifying pets and informing APHA by email rather than any automated data flow. Thirdly, the completion rate of case questionnaires declined since the introduction of self-completed online questionnaires, which may have resulted in a reduction in information of the number and/or type of pets in MPX case households. Fourthly, there may be other factors influencing potential under-reporting of case pet animal ownership to APHA/UKHSA which we have not yet been able to identify.

Importantly, MPXV infection may cause different clinical signs in animals compared with those observed in humans. International advice to pet owners has primarily been based on the symptoms displayed by humans considering the limited information on clinical presentation of MPX in non-human species [21]. If pet owners and veterinarians assessed the health of a potentially exposed animal based on these clinical signs alone, then there may have been MPX in pet animals which has gone undetected. There are also emerging reports of asymptomatic carriage in humans [22,23]. It is unknown at this stage whether non-human species have sub-clinical carriage, or if there is zoonotic transmission from asymptomatic animals, potentially contributing to overall human disease burden. Both the clinical profile of animal cases of MPX, and the potential for sub-clinical infection and transmission in non-human species, requires further research [24].

While there exists a risk of transmission to pets from owners with a confirmed MPXV infection, we assess this risk to be low [5]. However, if the virus were to spill over into peri-domestic rodents living in the UK, this could result in endemic foci for subsequent zoonotic transmission (and transmission to domesticated animals that could then infect their owners). UKHSA, APHA, and Defra will continue to monitor and assess for potential MPX cases in pets though the development of existing surveillance systems and communication with the public to raise awareness to prompt testing where indicated and ongoing research.

## Conclusions

Understanding the likelihood of reverse zoonotic transmission via pet animals associated with the current MPX outbreak will improve comprehension of the potential risk of further zoonotic ingress to the human population and inform further development of control measures to reduce spread – to both humans and other animals – via this route. Surveillance of pets in the households of confirmed human MPX cases is a valuable component to further our understanding of this risk.

## Supplementary Data

175000 Supplement
