# Supplementary material for: The risk of reverse zoonotic transmission to pet animals during the current global monkeypox outbreak, United Kingdom, June to mid-September 2022
Source: Euro Surveill. 2022 Sep 29;27(39):2200758. doi: 10.2807/1560-7917.ES.2022.27.39.2200758 (PMC9524050; doi:10.2807/1560-7917.ES.2022.27.39.2200758)
Supplement: Supplement [file 22-00758_SHEPHERD_Supplement.pdf]

This supplementary material is hosted by *Eurosurveillance* as supporting information alongside the article [Title] on behalf of the authors who remain responsible for the accuracy and appropriateness of the content. The same standards for ethics, copyright, attributions and permissions as for the article apply. Supplements are not edited by Eurosurveillance and the journal is not responsible for the maintenance of any links or email addresses provided therein.

S1: Assessing the risk of reverse zoonotic transmission to pets from the current global Monkeypox outbreak in the UK, 01 June to 16 September 2022

Table 1: Number and type of pets reported to APHA by household

# of animals in household	Dog	Cat	Rabbit or guinea pigs	Mammalian livestock	Other	Note
1	18	3			2	Unspecified + snake
2	3	10	1			
3		1	1			
4						
5	1					
6						
7				1		
8					1	Tropical frogs
9						
10						
11						
12						
13	1					
64					1	Poultry

Table 2: Total number of pets reported to APHA by number in household and pet type

# of animals in household	Dog	Cat	Rabbit or guinea pigs	Mammalian livestock	Other	Note
1	18	3			2	Unspecified + snake
2	6	20	2			
3		3				
4						
5	5					
6						
7				7		
8					8	Tropical frogs
9						
10						
11						
12						
13	13					
64					64	Poultry
